# Concealed expansion of immature precursors underpins acute burst of adult HSC activity in foetal liver

**DOI:** 10.1242/dev.131193

**Published:** 2016-04-15

**Authors:** Stanislav Rybtsov, Andrejs Ivanovs, Suling Zhao, Alexander Medvinsky

**Affiliations:** Institute for Stem Cell Research, Medical Research Council Centre for Regenerative Medicine, University of Edinburgh, SCRM Bioquarter, 5 Little France Drive, Edinburgh E16 4UU, UK

**Keywords:** AGM region, HSC, Embryo, Pre-HSC, Mouse

## Abstract

One day prior to mass emergence of haematopoietic stem cells (HSCs) in the foetal liver at E12.5, the embryo contains only a few definitive HSCs. It is thought that the burst of HSC activity in the foetal liver is underpinned by rapid maturation of immature embryonic precursors of definitive HSCs, termed pre-HSCs. However, because pre-HSCs are not detectable by direct transplantations into adult irradiated recipients, the size and growth of this population, which represents the embryonic rudiment of the adult haematopoietic system, remains uncertain. Using a novel quantitative assay, we demonstrate that from E9.5 the pre-HSC pool undergoes dramatic growth in the aorta-gonad-mesonephros region and by E11.5 reaches the size that matches the number of definitive HSCs in the E12.5 foetal liver. Thus, this study provides for the first time a quantitative basis for our understanding of how the large population of definitive HSCs emerges in the foetal liver.

## Abstract (precis)

**Summary:** A quantitative approach reveals how the pre-HSC pool undergoes dramatic growth in the AGM region and, by E11.5, reaches the size that matches the number of definitive HSCs in the E12.5 foetal liver.

## INTRODUCTION

Although the first signs of haematopoietic differentiation in mouse development are observed in the embryonic day (E) 7.5 yolk sac of the mouse embryo, definitive HSCs (dHSCs), which generate the adult haematopoietic system and are detectable by direct transplantation into adult irradiated recipients, emerge only by E10.5-E11.5 in very small numbers (Kumaravelu et al., 2002; Medvinsky and Dzierzak, 1996; Müller et al., 1994). However, by E12.5 the foetal liver contains approximately 50-60 fully functional dHSCs (Ema and Nakauchi, 2000; Kumaravelu et al., 2002). The cellular processes underlying this dramatic rapid appearance of dHSC activity in the foetal liver remain obscure.

Given that dHSCs that repopulate adult irradiated recipients appear only by the end of E10, it is broadly understood that dHSCs develop from immature HSC precursors (pre-HSCs), which are not detectable by direct transplantation (Medvinsky and Dzierzak, 1996). However, the quantitative anatomy and dynamics of growth of this embryonic rudiment of the adult haematopoietic system, is not characterised.

In the E11.5 mouse embryo, the aorta-gonad-mesonephros (AGM) region, placenta, extra-embryonic arteries, yolk sac and head contain no more than one dHSC per tissue (de Bruijn et al., 2000; Gekas et al., 2005; Gordon-Keylock et al., 2013; Kumaravelu et al., 2002; Li et al., 2012; Mikkola et al., 2005; Müller et al., 1994; Ottersbach and Dzierzak, 2005; Robin et al., 2009; Yoder et al., 1997). Although various lines of evidence demonstrate a primary role of the AGM region in different vertebrate species in HSC development (Ciau-Uitz et al., 2014; Clements and Traver, 2013; Dieterlen-Lievre, 1975; Dzierzak and Speck, 2008; Galloway and Zon, 2003; Ivanovs et al., 2013, 2014, 2011; Medvinsky et al., 2011, 1993; Müller et al., 1994), the contribution of other tissues into the pool of adult HSCs is less clear. A key question regarding the contribution of different tissues is which of them contain pre-HSCs and, for those that do, how big is this population?

It has been possible to identify functionally the presence of pre-HSCs by maturing pre-HSCs *ex vivo* (Medvinsky and Dzierzak, 1996; Rybtsov et al., 2011; Taoudi et al., 2008). Apart from the AGM region, only large extra-embryonic arteries have been shown to possess the potential to generate definitive HSCs *de novo*, although to a lesser extent (de Bruijn et al., 2000; Gordon-Keylock et al., 2013; Medvinsky and Dzierzak, 1996; Taoudi et al., 2008). Previous experiments showed that the AGM region is a potent source of HSCs. Although previously we and others demonstrated quantitatively the massive dHSC outcome in culture, that approach did not enable information about the initial (input) numbers of HSC precursors (pre-HSCs) isolated from embryonic tissues to be obtained (Hadland et al., 2015; Rybtsov et al., 2011; Taoudi et al., 2008).

**Fig. 1.**
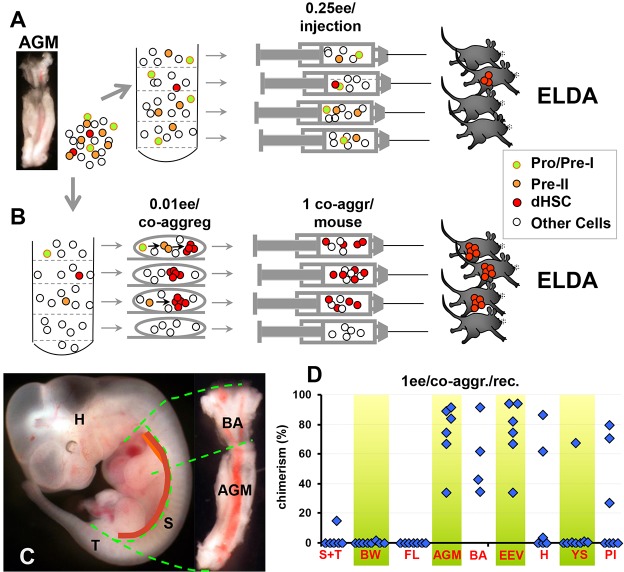
**Comparative limiting dilution analysis of pre-HSC sites.** (A) Standard limiting dilution analysis for dHSCs using direct transplantations of freshly isolated cells. (B) Pre-HSC limiting dilution assay (preHSC-LDA). AGM cell suspension contains pro-HSCs and Type I pre-HSCs (green circles), Type II pre-HSCs (orange circles) and dHSCs (red circles). Each whole co-aggregate was injected in an individual recipient (one co-aggregate per mouse). Note that 0.01 ee dose for co-aggregation is indicated as an example and varied in actual experiments. For further description, see Materials and Methods. (C) Dissection boundaries (green dashed lines) of cultured and transplanted tissues (E11.5). Position of the dorsal aorta (including bifurcated rostral part) is shown by thick orange line. (D) Tissue distribution of pre-HSCs. Each isolated tissue (as indicated in C) was co-aggregated individually with OP9 cells, cultured and each co-aggregate transplanted individually into a recipient (1 ee/co-aggregate/recipient). Blue diamonds represent donor-derived blood chimerism (%) in individual mice. Numbers of non-repopulated mice were used for quantification of pre-HSC as described in Materials and Methods for preHSC_LDA. AGM, aorta-gonad-mesonephros; BA, bifurcated dorsal aorta; BW, body wall; EEV, extra-embryonic vessels; FL, foetal liver; H, head; Pl, placenta; S, somites; T, tail; YS, yolk sac.

Here, we have sought confirmation that the size of the *in vivo* pre-HSC pool is large enough to build the dHSC pool in the foetal liver in less than 24 h. To this end, we designed a limiting dilution approach to assess quantitatively the size of the pool of immature HSC precursors in embryonic tissues *in vivo*, using an *ex vivo* system (Fig. 1B).

Previous functional analysis identified VE-cadherin^+^ (cadherin 5) cells, which sequentially upregulate CD41 (Itga2b), Runx1 (AML1), CD43 (Spn) and CD45 (Ptprc) haematopoietic markers, as *in vivo* embryonic precursors of dHSCs (Chen et al., 2009; Liakhovitskaia et al., 2014; Rybtsov et al., 2014, 2011). HSC maturation from the endothelial compartment occurs through a four-step process: pro-HSC→Type I pre-HSC→Type II pre-HSC→dHSCs (Rybtsov et al., 2014, 2011; Taoudi et al., 2008). Pro-HSCs were defined as VE-cad^+^CD41^+^CD43^−^CD45^−^ cells, Type I pre-HSCs as VE-cad^+^CD41^low^CD43^+^CD45^−^ cells and Type II pre-HSCs as VE-cad^+^CD41^low^CD43^high^CD45^+^ cells. dHSCs have the same phenotype as Type II pre-HSCs (VE-cad^+^CD41^−/low^CD43^high^CD45^+^), but can be assayed by direct transplantation into adult irradiated recipients. All four types of pre-HSCs have been identified morphologically in the area of the dorsal aorta (Rybtsov et al., 2014, 2011; Taoudi et al., 2008; Yokomizo et al., 2011).

Here, using a novel quantitative approach (Fig. 1B; see also Materials and Methods), we unveil for the first time an otherwise concealed dramatic expansion of immature precursors that lay the foundation of the adult haematopoietic system. Our data strongly suggest that by E11.5 the size of the pre-HSC population in the AGM region is sufficient to form the large dHSC pool in the foetal liver through a quick maturation step.

## RESULTS

### Limiting dilution approach to quantify pre-HSCs

Although dHSCs have previously been quantitatively mapped in the embryo using limiting dilution analysis (Fig. 1A) (Ema and Nakauchi, 2000; Kumaravelu et al., 2002; Sutherland et al., 1990), the numbers of pre-HSCs, which produce dHSCs *in vivo* in these tissues, remained unknown. Here, we designed an *ex vivo* approach to quantify pre-HSCs *in vivo*, which is based on a culture system that allows all HSC precursor types to mature into fully functional dHSCs (Rybtsov et al., 2014). Because pre-HSCs can only be detected after a period of *ex vivo* culture, we diluted the pre-HSC population to limiting dilution prior to *ex vivo* culture and transplantation.

Specifically, cell suspensions obtained from dissected embryonic tissues were divided into portions, each separately co-aggregated with OP9 cells and cultured in conditions supporting pre-HSC maturation. Importantly, our experimental design excludes any contribution of cell proliferation (either by pre-HSCs or dHSCs) into calculations of pre-HSC numbers. Namely, cultured co-aggregates were transplanted individually into adult irradiated recipients (one co-aggregate per mouse). In these circumstances, any derivatives of a maturing pre-HSC or dHSCs remained contained within their original co-aggregates and injected into an individual recipient. Thus, if pre-HSC or dHSC expansion occurs in culture, it is constrained by each co-aggregate, and does not influence the number of positive co-aggregates. This allowed us to determine retrospectively which co-aggregates contained no pre-HSCs (i.e. gave no repopulation) and those which contained at least one pre-HSC (i.e. gave repopulation) prior to culture (Fig. 1B). The proportion of mice repopulated is used to count the number of pre-HSCs in the initial undiluted (*in vivo*) population using extreme limiting dilution analysis (ELDA; Hu and Smyth, 2009). For conciseness, this assay will be termed here pre-HSC limiting dilution assay (preHSC_LDA) (Fig. 1B; see also Materials and Methods). Where the term pre-HSC is used in this paper, we are referring to any of our previously defined non-repopulating precursor cell types (pro-HSC, Type I pre-HSC and Type II pre-HSC).

### Identification of major sites of pre-HSC activity prior to liver colonisation

**Fig. 2.**
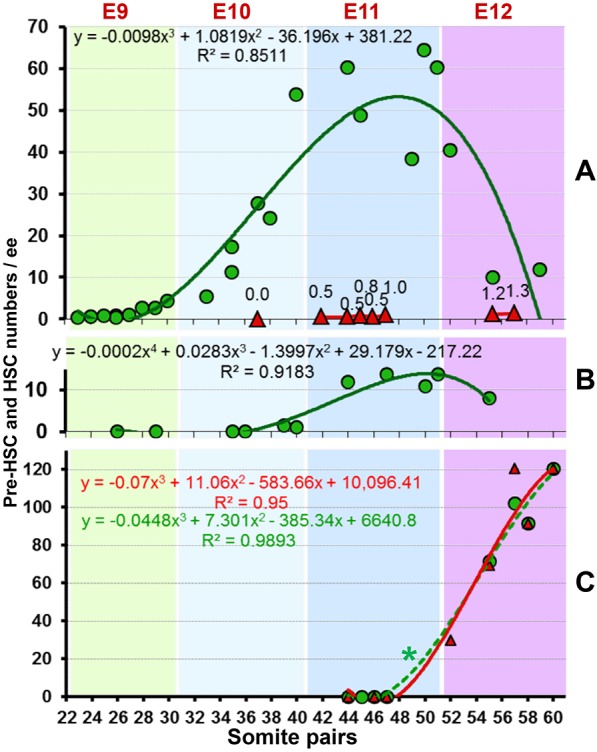
**Quantitative developmental dynamics of pre-HSCs and HSCs in early embryo.** (A-C) Dynamics of pre-HSC (green) and dHSC (red) numbers in caudal part/AGM region (A), extra-embryonic vessels (B) and foetal liver (C). Predicted polynomial regression curve, equation and correlation (R^2^) for pre-HSCs (green) and dHSCs (red) for each tissue are shown. Days post-fertilisation are indicated along the top and somite counts are along the lower axis. Each symbol represents a pre-HSC/dHSC number obtained from an individual experiment. Note that each symbol represents pre-HSC or HSC number in an individual experiment (not in an individual animal) calculated by preHSC_LDA (in green) or dHSC LDA (in red; as assayed by direct transplantations of fresh tissues); for methodology see Fig. 1B and 1A, respectively. For example, the curve for pre-HSCs in the AGM region represents 27 experiments. Full summary of experiments with repopulation levels of individual mice is presented in Table S2. Note that green dashed line (marked by asterisk) (C), represents pre-existing dHSCs in the foetal liver, which are maintained in culture, as numbers coincide with those calculated by direct transplantations shown in red (for detailed explanation, see Results).

In our initial experiments, preHSC_LDA showed that the E11 AGM region, together with the anterior trunk containing the bifurcated dorsal aorta (BA) and extra-embryonic vessels (EEVs), are enriched for pre-HSCs (Fig. 1C,D). Indeed, all recipients transplanted with whole individual co-aggregates containing one embryo equivalent (ee) of the embryonic tissue were repopulated at high level with donor-derived haematopoietic cells. Statistically, this means that each E11.5 AGM region contained at least one pre-HSC. More precise estimates derived from further dilutions will be described in the next section. By contrast, transplantations of co-aggregates with 1 ee of other HSC niches, such as placenta (Pl) and head (H), gave a proportion of non-repopulated mice (Fig. 1D), which allowed us to apply preHSC_LDA and calculate that the E11.5 placenta contains one or two pre-HSCs and the head contains zero or one pre-HSC per embryo (Fig. S2B,D). Notably, we observed a tendency of increasing pre-HSC numbers by the end of E11 in the placenta, in line with previous observations of placental dHSC dynamics (Gekas et al., 2005). In all cases, long-term multi-lineage repopulation was observed (Fig. S1A). Practically no pre-HSCs were detected in somites and tail (S+T), body walls (BW), foetal liver (FL) or yolk sac (YS) carefully separated from EEVs (Fig. 1D). Therefore, pre-HSCs in the E11.5 embryo are predominantly localised to the AGM region (Figs 1 and 2; see also Fig. S2B-D). Saturated repopulation (lack of non-repopulated mice) with the AGM region, BA and EEVs in these experiments does not allow preHSC_LDA to be used for precise assessment of pre-HSC numbers in these tissues (Fig. 1D). To this end, higher cell dilution doses have been used as described below. Because both the AGM region and the more rostrally located BA are significantly enriched for pre-HSCs (Fig. 1C,D), further investigations of these anatomically linked parts were performed as a single entity.

### Pre-HSC pool expands rapidly between E9.5 and E11.5

To investigate developmental dynamics of pre-HSC expansion *in vivo*, we assessed pre-HSC numbers at several developmental time points (stages) from E9 to E12.5 using preHSC_LDA (Fig. 2; Table S2). Embryos were staged according to the number of somite pairs (sp). We found that the E9.5 caudal part contains no more than one or two pro-HSCs (Fig. 2A). By E10 we observed a steady and rapid increase in pre-HSC numbers from approximately five at early E10 up to 20-30 by mid-E10 (35 sp) and ∼50 by the end of E10 (39 sp). During E11 pre-HSC numbers reached a peak of 65 cells and by E12.5 the number of pre-HSCs dramatically dropped to about ten, likely reflecting their shift to the foetal liver (Fig. 2). Pre-HSCs in extra-embryonic arteries begin to emerge from late E10.5, and by E11 reach 15 cells (Fig. 2B). Therefore, our estimates show that by late E11 the embryo contains ∼70 pre-HSCs in total (54 preHSCs and 15 preHSCs in the AGM region and EEV, respectively). Comparable numbers of pre-HSCs at E11.5 were obtained in two additional limiting dilution experiments with whole caudal parts and extra-embryonic vessels (Fig. S2A).

We found that the E11.5 foetal liver entirely lacks pre-HSC or dHSC activity (Fig. 2C). By the following day, the number of dHSC in the foetal liver (as assessed by direct transplantation) is 30 and reaches 120 by late E12. Although low level repopulating activity was reported in *Rag*2*γc*^−/−^ mice from an earlier foetal liver that was cultured in the presence of thrombopoetin on OP9 cells (Kieusseian et al., 2012), the relation of these cells to the definitive HSC lineage remains unclear. Of note, in the E12.5 foetal liver numbers of cells calculated by the preHSC_LDA over sequential somite pair stages closely coincide with respective dHSCs numbers assessed by direct transplantations.

Because dHSCs are supported in these culture conditions (as tested on adult bone marrow HSCs, data not shown) their numbers also contributed to preHSC_LDA estimates. Therefore, to obtain actual numbers of pre-HSCs, dHSC numbers detected *in vivo* by direct transplantations have to be subtracted from preHSC_LDA estimates. Such mathematical procedure does not change significantly pre-HSC numbers in the AGM region owing to absence or low numbers of dHSCs. However, in the foetal liver numbers of dHSCs (Fig. 2C, red line) and those obtained by preHSC_LDA (Fig. 2C, dashed green line) coincide and therefore, by deduction, this strongly suggests that the E12.5 liver contains only dHSCs and no pre-HSCs.

Remarkably, the number of pre-HSCs in E11.5 AGM region assessed by preHSC_LDA coincides with dHSC numbers in the E12.5 foetal liver (identified by direct transplantations). Thus, despite the considerable presence of VE-cad^+^ cells in the foetal liver, they are not related to dHSCs, in line with the view that the liver is colonised from outside.

The quantitative matching of pre-HSC numbers in the AGM region and dHSC numbers in the foetal liver allows us to conclude with high confidence that the *ex vivo* HSC maturation system does not artificially stimulate dHSC generation from cells outside the pre-HSC lineage. However, this quantitative matching also suggests that practically all immature pre-HSCs are captured, strongly indicating that the *ex vivo* assay employed here adequately identifies pre-HSC populations *in vivo*.

### Composition dynamics of the developing HSC pool

**Fig. 3.**
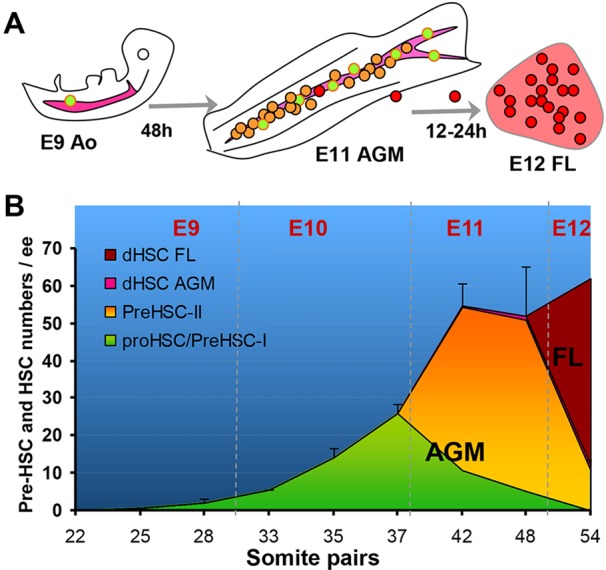
**Dynamic composition of pre-HSC/dHSC development.** (A) Growing pool of pre-HSCs in the AGM region builds up by E11.5 and rapidly colonises the foetal liver (FL) by E12.5 (during 12-24 h) (cells are colour coded as in Fig. 1). Ao, dorsal aorta. (B) Cumulative number of pro-HSCs and Type I pre-HSCs (green area) relative to more mature Type II pre-HSCs (orange) and dHSCs (pink), which appear and expand at later stages in the AGM region. Numbers of AGM-derived pre-HSCs match dHSC numbers in E12 foetal liver (brown). Foetal liver is represented by brown area; the AGM region is represented by green, orange and pink areas. Each experimental point is the mean of three or four experiments (standard deviations are shown) and derived from Table S2 (calculated using preHSC_LDA). Ratio between pre-HSC Type I and II derived from Table S1 and Fig. S1B,C.

We next investigated the composition dynamics of the developing HSC hierarchy throughout E9.5-E12.5 stages. Fig. 3A,B illustrates pre-HSC development in the AGM region followed by accumulation of the dHSC pool in the foetal liver. The ratio between the earliest CD45^−^ (pro-HSC and Type I pre-HSC) and more advanced CD45^+^ (Type II pre-HSC) embryonic precursors was assessed. To this end, we sorted E10.5 VE-cad^+^CD45^−^ and VE-cad^+^CD45^+^ populations and co-aggregated them with OP9 stromal cells (Fig. S1B). After culture, co-aggregates were pooled and transplanted into irradiated recipients, confirming that 30-38 sp (E10) AGM regions contain only CD45^−^ HSC precursors. We found that Type II pre-HSCs appear only after the end of E10 (Fig. S1B) (Rybtsov et al., 2011). At E11.5, the AGM region still contains Type I pre-HSCs, but also develops Type II pre-HSCs (Rybtsov et al., 2011). To evaluate the ratio between Type I pre-HSCs and Type II pre-HSCs at this stage, these populations were sorted and co-aggregated with OP9 cells in limiting dilutions. Using preHSC_LDA (Fig. 1B), we established that the quantitative Type I to Type II pre-HSC ratio is 1:4 (all embryos are 41-45 sp; *n*=6 experiments) (Table S1). As the average number of pre-HSCs in the AGM region at this stage is 54±5.8 (Fig. 3B; Table S2), the quantitative composition of the HSC hierarchy (pre-HSC Type I: pre-HSC Type II: dHSC) is 11:42:1, given the presence of approximately one dHSC as detected by direct transplantation (Fig. 2A) (Kumaravelu et al., 2002; Taylor et al., 2010). By E12, the AGM region still contains Type II pre-HSCs; however, Type I pre-HSCs are no longer detectable, suggesting that *de novo* pre-HSC formation is finished (Fig. S1C).

## DISCUSSION

Previous reports using newborn recipients indicated the presence of 1-12 pre-HSCs in the E10-E11 AGM region (Arora et al., 2014; Boisset et al., 2014). Our study gives significantly higher numbers, which could be explained by: (1) use of the highly controlled and efficient *ex vivo* HSC maturation system and (2) robust homing of mature dHSCs into adult recipients, compared with engraftment of immature pre-HSCs in newborn animals. As single adult bone marrow HSCs can engraft at a very high frequency of 70-96% upon intravenous injections (Kiel et al., 2005; Matsuzaki et al., 2004; Wilson et al., 2008), this strongly indicates that the number of their counterpart dHSCs matured from embryonic pre-HSCs in the culture, is accurately estimated by intravenous transplantations.

The expansion of the pre-HSC population found here during embryo development, in principle, can occur through their proliferation as well as maturation from a more immature subset of cells. Further development of experimental approaches will be required to discriminate between these two scenarios.

At which anatomical location does the conversion of pre-HSCs into dHSCs occur? At E11 we do not see active dHSC maturation in the AGM region, but at E12 the liver is already full of dHSCs. Several scenarios are possible: (1) although the first dHSCs appear in the AGM region, sequential maturation of the following ones occur followed by immediate relocation into the foetal liver (in this case, large numbers of dHSCs would never be seen in the AGM region but will gradually increase in the foetal liver); (2) a short synchronous pulse of dHSC maturation inside the AGM region occurs sometime between E11 and E12, e.g. during night time, but this would be practically impossible to capture experimentally; (3) pre-HSCs can gradually migrate through EEVs to the placenta where they mature and subsequently relocate to the foetal liver. As an indication for the third possibility, we see a tendency for pre-HSC numbers in EEVs to increase towards the end of E11 (Fig. 2C). This attractive possibility may link our data to previously published studies (Gekas et al., 2005; Ottersbach and Dzierzak, 2005). The first two scenarios imply that dHSC maturation occurs in the AGM region prior to liver colonisation and would be in line with the previous report showing that contact with the liver is not essential for HSC maturation (Taoudi et al., 2005). Which scenario actually takes place will need to be clarified in future studies.

In summary, our data show that between E9.5 and E11.5 the pool of HSC precursors gradually matures and expands dramatically predominantly in the AGM region and at lower levels in extra-embryonic arteries. By E11.5, the size of the pre-HSC pool remarkably matches the number of mature HSCs appearing within less than 24 h in the foetal liver. Although some other embryonic tissues also contain pre-HSCs, their numbers are negligible compared with the AGM region. This finding offers a simple explanation for the abrupt appearance of the large pool of definitive HSCs in the foetal liver from pre-HSCs through a short maturation step, which in principle does not require any significant proliferation. The transition from the AGM region to the liver stage is reflected by a rapid decrease of pre-HSCs in the AGM region from 40-65 to ten, concurrent with a dramatic rise in the mature dHSC pool in the foetal liver between E11.5 and E12.5 (Fig. 2A,C). Thus, this study unveils for the first time a unique, previously unidentified cellular process that underlies the dynamic growth of the adult haematopoietic system rudiment in early embryogenesis.

## MATERIALS AND METHODS

### Animals

Staged embryos were obtained by crossing C57BL/6 (CD45.2/2). The morning of discovery of the vaginal plug was designated as E0.5. Accurate embryo staging was performed by counting somite pairs (sp) and grouping as follows: E9.0 (15-23 sp), E9.5 (24-30 sp), E10.0 (31-34 sp), E10.5 (35-39 sp), E11.0 (40-45 sp) E11.5 (46-49 sp), E12.0 (51-55 sp), E12.5 (<56 sp). All experiments using animals were performed under a Project Licence granted by the Home Office (UK), approved by the University of Edinburgh Ethical Review Committee, and conducted in accordance with local guidelines.

### Long-term repopulation assay

Wild-type C57BL/6 CD45.2/2 cells were injected intravenously into adult γ-irradiated CD45.1/2 heterozygous recipients [split dose (471+471 Rad) with 3 h interval] along with 100,000 CD45.1/1 nucleated bone marrow carrier cells per recipient.

Number of cells injected was expressed in embryo equivalents (ee). For sorted cells, ee values were adjusted for percentage of dead cells. For quantification of dHSC numbers, embryonic tissues isolated from different developmental stages were dissected, dissociated and injected directly in limiting dilutions into cohorts of recipients (Fig. 1A). Donor-derived chimerism (%) was evaluated in peripheral blood at 6.5 weeks and 13.5-18 weeks post-transplantation using a FACSCalibur or Fortessa (BD Biosciences) (our previous observations on >30 recipients of early embryo-derived dHSCs matured in culture showed that repopulation at 13.5 weeks persists at least until week 18). Erythrocytes were depleted using PharM Lyse (BD Biosciences) and cells were stained with anti-CD16/32 (Fc-block) followed by anti-CD45.1-APC (clone A20, cat. #12-0454-83) and anti-CD45.2-PE (clone 104, cat. #17-0453-82) monoclonal antibodies (eBioscience). Multi-lineage donor-derived haematopoietic contribution in recipient blood and organs were determined by staining with anti-CD45.1-V450 (cat. #560520), anti-CD45.2-V500 (cat. #562129) and lineage-specific anti-Mac1 FITC (cat. #553310), Gr1-PE (cat. #553128) CD3e-APC (cat. #553064), B220-PE-Cy7 (cat. #552772) monoclonal antibodies (BD Pharmingen). We defined dHSCs as cells with long-term multi-lineage repopulation capacity with chimerism >5% in recipient peripheral blood.

### E9.5-E12.5 embryo dissection

Embryonic tissues were dissected as previously described for the AGM region (Rybtsov et al., 2014, 2011; Taoudi et al., 2008), for extra-embryonic vessels and yolk sac (Gordon-Keylock et al., 2013), for the placenta (Gekas et al., 2005; Ottersbach and Dzierzak, 2005) and for the head (Li et al., 2012). E11 AGM was dissected together with the BA (Fig. 1C).

### Fluorescence-activated cell sorting and analysis

The following antibodies were used: anti-CD45 FITC (fluorescein isothiocyanate; clone 30-F11, cat. #553080); anti-mouse VE-cadherin biotin-conjugated antibody (clone 11D4.1, cat. #555289) following streptavidin APC staining (all from BD Pharmingen). Cells were sorted using a FACSAria-II sorter (BD Biosciences) followed by purity checks as described before (Rybtsov et al., 2011). Gating of negative populations was performed on the basis of fluorescence minus one (FMO) staining where one of the antibodies was replaced with an isotype control (IC) (BD Pharmingen). Dead cells were excluded by 7AAD staining. Data acquisition and data analysis was performed by Fortessa (BD Biosciences) using FlowJo software (Tree Star).

### PreHSC_LDA

At the core of this method is the co-aggregation culture system, which allows maturation of HSC precursors. In these culture conditions, all types of HSC precursors isolated from E9.5-E11.5 embryos can mature into dHSCs (Rybtsov et al., 2014). However, this approach does not allow pre-HSCs to be quantified. Previously the limiting dilution approach (LDA) has successfully been used for calculation of numbers of mature adult haematopoietic stem cells (Fig. 1A) (Szilvassy et al., 2002).

Our novel approach, termed preHSC_LDA, is designed here to assess pre-HSC numbers present *in vivo* (Fig. 1B). For this purpose, dissociated tissues were divided into portions to achieve the appropriate dilution (0.01-1.00 ee); each portion was co-aggregated separately with 10^5^ OP9 cells and allowed to mature in culture. Based on the principle of quantitative limiting dilution analysis, we have to use doses of cells sufficiently low to obtain some statistically significant numbers of co-aggregates with and without pre-HSCs. As a result, by the end of culture, some co-aggregates will contain and some will not contain dHSCs. Transplanting individual co-aggregates into separate recipients gave us the number of co-aggregates that originally (before culture) contained pre-HSCs, independently of their proliferative activity inside the co-aggregate. This is because all the progeny of the pre-HSC remains in the same co-aggregate and goes only into one recipient mouse. Using Poisson's statistics (ELDA software; Hu and Smyth, 2009) based on numbers of repopulated versus total transplanted recipients, we could calculate the number of pre-HSCs in a given embryonic tissue.

For culture, E9.5-E12.5 embryonic tissues were dissociated with collagenase/dispase and then co-aggregated in limiting dilutions with 10^5^ OP9 cells using 30 μl culture medium [Iscove's modified Dulbecco's medium (IMDM; Invitrogen-GIBCO), 20% of preselected, heat-inactivated foetal calf serum (PAA/HyClone) and L-glutamine, penicillin/streptomycin]. Cells were centrifuged at 450 ***g*** for 12 min in 200 μl pipette tips sealed with parafilm as described previously (Rybtsov et al., 2014; Sheridan et al., 2009). Media was supplemented with growth factors expressed in the AGM region [SCF (Kitl), Flt3, IL3 at 100 ng/ml each; PeproTech]. Co-aggregates were cultured on floating 0.8-μm AAWP 25-mm nitrocellulose membranes (Millipore) for 24 h, then medium was replaced and cultures incubated for an additional 6 days. Co-aggregates were dissociated using collagenase/dispase. All experiments were performed at least twice independently.

Throughout this study, pre-HSC_LDA was mainly used without distinction (no cell purification) between types of pre-HSCs (Fig. 1D; Fig. 2). However, when quantification of individual pre-HSC types was required, we sorted these populations and subjected them to preHSC_LDA (Table S1). Mice with donor-derived chimerism >5% following 13.5-18 weeks post-transplantation were considered to be repopulated (Table S2).

### Statistical analysis

The number of pro-HSCs and HSCs and the 95% confidential interval were calculated by the single-hit Poisson model using ELDA software (Hu and Smyth, 2009). Coefficients for polynomial regression equation were calculated in Excel (Microsoft). Polynomial regressions were calculated and close correlation with experimental data (R^2^ is 0.84-0.98) (*P*>0.01) were found (*x*-axis: somites pairs number; *y*-axis: pre-HSC/HSC number) (Fig. 2; Table S2).
